# Logistic regression analysis of conventional ultrasonography, strain elastosonography, and contrast-enhanced ultrasound characteristics for the differentiation of benign and malignant thyroid nodules

**DOI:** 10.1371/journal.pone.0188987

**Published:** 2017-12-11

**Authors:** Tiantian Pang, Leidan Huang, Yingyuan Deng, Tianfu Wang, Siping Chen, Xuehao Gong, Weixiang Liu

**Affiliations:** 1 Health Science Center, Shenzhen University, Shenzhen 518060, China; 2 School of Biomedical Engineering, Shenzhen University, Shenzhen 518060, China; 3 National-Regional Key Technology Engineering Laboratory for Medical Ultrasound, Shenzhen 518060, China; 4 Guangdong Key Laboratory for Biomedical Measurements and Ultrasound Imaging, Shenzhen 518060, China; 5 Department of Ultrasound, First Affiliated Hospital of Shenzhen University, Second People’s Hospital of Shenzhen, Shenzhen 518035, China; 6 Guangzhou Medical University, Guangzhou 510182, China; Nanjing University, CHINA

## Abstract

The aim of the study is to screen the significant sonographic features by logistic regression analysis and fit a model to diagnose thyroid nodules. A total of 525 pathological thyroid nodules were retrospectively analyzed. All the nodules underwent conventional ultrasonography (US), strain elastosonography (SE), and contrast -enhanced ultrasound (CEUS). Those nodules’ 12 suspicious sonographic features were used to assess thyroid nodules. The significant features of diagnosing thyroid nodules were picked out by logistic regression analysis. All variables that were statistically related to diagnosis of thyroid nodules, at a level of *p* < 0.05 were embodied in a logistic regression analysis model. The significant features in the logistic regression model of diagnosing thyroid nodules were calcification, suspected cervical lymph node metastasis, hypoenhancement pattern, margin, shape, vascularity, posterior acoustic, echogenicity, and elastography score. According to the results of logistic regression analysis, the formula that could predict whether or not thyroid nodules are malignant was established. The area under the receiver operating curve (ROC) was 0.930 and the sensitivity, specificity, accuracy, positive predictive value, and negative predictive value were 83.77%, 89.56%, 87.05%, 86.04%, and 87.79% respectively.

## Introduction

In clinical practice, thyroid nodules have a high incidence. The incidence in the general population is approximately 4%-7% by palpation. With widespread use of high frequency ultrasonography (US), the prevalence of thyroid has risen to 67% [1]. The most thyroid nodules are benign, while less than 10% are malignant [2, 3]. Therefore, the differentiation of benign and malignant thyroid nodules is full of challenges. There are various malignant sonographic features but lack of consistent criterion. Some scholars have proposed the concept of Thyroid Imaging Reporting and Data system (TI-RADS) which refers to the Breast Imaging Reporting and Data system (BI-RADS) [3]. Horvath et al. first proposed TI-RADS in 2009 which defined 6 categories based on 10 sonographic features, including shape, orientation, echogenicity, echo structure, acoustic transmission, borders, surface, presence or absence of a capsule, calcifications, and vascularization. Park et al. established TI-RADS and developed 5 categories based on sonographic features. The study indicated that characteristics such as taller-than-wide shape, a not circumscribed margin, solid composition, homogeneous echotexture, marked hypoechogenecity, hypoechogenecity, presence of microcalcification, absence of a perinodular halo, and presence of an adjacent abnormal lymph node predicted a malignant classification [4]. Strain elastography (SE) is a new dynamic technology to evaluate the elasticity of tissue qualitatively by exerting external force. The hard tissue is less distortion than the soft in the same situation. Different degree of distortion is used to examine the hardness of tissue. Based on the fact that malignant thyroid nodules are harder than benign, SE is used to distinguish benign and malignant nodules [5, 6]. CEUS is a technique that infuses micro-bubble to blood capillary which is smaller than erythrocyte. Due to the ultrasound scattering effect produced by blood capillary, it can estimate the blood perfusion features of thyroid nodules to evaluate the angiogenesis situation [7]. Accuracy of diagnosis for thyroid nodules can be increased by combining CEUS and conventional US [8].

Relevant published articles are consistent with the finding that irregular margin, ill-defined border, taller-than-wide shape, hypoechogenicity or marked hypoechogenicity, microcalcification and so on are the US suspicious features of malignant thyroid nodules [9]. Up to now, various studies performed TI-RADS to differentiate benign and malignant thyroid nodules and identify whether it has a good diagnostic performance of thyroid lesions or not. However, the existing studies have not covered elastography and angiography parameters. Based on the previous researches, this study intends to establish a model and to evaluate the fitted model’s diagnostic performance in the determination of thyroid lesions by logistic regression in 12 clinical sonographic features from US, SE, and CEUS.

## Materials and methods

### Patients

The ethics committee of Shenzhen Second People’s Hospital approved this study. Written consent was obtained from all the patients. We retrospectively reviewed database (S1 Data) of 525 thyroid nodules from 525 patients (average age of 44 ± 11 years; 129 male and 396 female) who underwent conventional US, SE, and CEUS from March 2014 to December 2016. All the thyroid nodules were pathologically proven.

### Sonographic examinations and characteristics

S2000 US scanner (Siemens Medical Solutions, Mountain View, CA, USA) equipped with 9L4 transducer (bandwidth frequency of 4–9 MHz) and acoustic radiation force impulse (ARFI) software was used to perform the sonographic examinations. The conventional US, SE, and CEUS examinations were performed by one radiologist with at least 10 years of experience. Patients were kept in a supine position with the neck fully exposed. All the sonographic images were review by 2 radiologists with 10-year experience in thyroid blindly. When discrepancy occurred, agreement was reached after discussion.

Firstly, all the patients underwent conventional US, involving B mode US and color Doppler US. Conventional US features include: margin, border, shape, echogenicity, calcification, posterior acoustic, peripheral acoustic halo, capsule of thyroid, vascularity, and suspected cervical lymph node metastasis. Margin was categorized as regular or irregular. Border was classified as well-defined (obvious boundaries between the nodule and surrounding thyroid parenchyma) or ill-defined. Shape was classified as taller-than-wide or wider-than-taller. The echogenicity of thyroid nodule was categorized as hyperechogenicity, isoechogenicity, hypoechogenicity compared with normal thyroid parenchyma, or marked hypoechogenicity when the lesion was hypoechoic relative to the neighboring strap muscles. In addition, the presence of hyperechogenicity and isoechogenicity were regard as no hypoechogenicity or no marked hypoechogenicity in this study. Presence of calcification was classified as microcalcification (<1*mm* in size; punctuate, small intranodular hyperechoic without posterior acoustic shadows) or no microcalcification (coarse calcifications or no calcification). The posterior acoustic of nodule was categorized as posterior echo attenuation, no findings, or posterior echo enhancement. Additionally, the presence of posterior echo enhancement and no finding were regard as no posterior echo attenuation in this study. The acoustic halo at the peripheral of thyroid nodule was classified as presence or absence. The capsule of thyroid was classified as interruption or completeness. Vascularity was categorized as no vascularity (no color Doppler flow within the nodule and at the peripheral), peripheral vascularity (color Doppler flow at the peripheral of the nodule and no color Doppler flow within the nodule), and central vascularity (color Doppler flow within the nodule). In addition, no vascularity and peripheral vascularity were regarded as no central vascularity in this study. Lymph nodes with the absence of hyperechoic hilum and the nodes with irregular shape was regarded as suspected cervical lymph node metastasis.

ARFI-induced SE examination was carried out following conventional US. The ARFI-induced SE image could reflect the elasticity of tissue in the field of view (FOV) by grayscale over the conventional B-mode image. The score for SE was referred to Xu’s scoring system [10]: score 1, the nodule is showed as predominantly white; score 2, the nodule is showed as predominantly white with few black portions; score 3, the nodule is showed as equally white and black; score 4, the nodule is showed as predominantly black with a few white spots; score 5, the nodule is showed as almost completely black; score 6, the nodule is showed as completely black without white spots. The nodule was regarded as malignant if the score is greater than 4.

CEUS was performed with contrast pulse sequencing (CPS) ultrasound imaging mode. The contrast agent used was SonoVue (Bracco S.p.A Inc., Milan, Italy). Intravenous access was established by elbow vein. A total of 25*mg* contrast medium was dissolved in 5*ml* 0.9% sodium chloride. Subsequently, it was injected as an intravenous bolus of 1.2*ml* per subject through the elbow vein, followed by a 5*ml* normal saline flush. Under the contrast mode, the mechanical index (MI) ranged between 0.05 and 0.07. The focus was adjusted to the lower edge or the middle of the thyroid nodule. The continuous dynamic images were recorded for a minimum of 2 minutes on the machine’s internal hard drive for the purpose of analyzing off-line. With the comparison of echogenicity brightness between thyroid nodule and surrounding parenchyma at peak enhancement, the degree of enhancement was classified as hypo-enhancement, iso-enhancement, hyper-enhancement and no-enhancement. According to the identity of echogenicity intensity of thyroid nodule, the enhancement identity was classified as homogeneous and heterogeneous. Additionally, the nodule was regarded as malignant if the pattern of enhancement is heterogeneous hypo-enhancement.

In this study, we evaluated the diagnostic value of the following 12 sonographic features to differentiate malignant and benign thyroid nodules. Sonographic features included margin (*X*_1_), border (*X*_2_), shape (*X*_3_), echogenicity (*X*_4_), calcifications (*X*_5_), posterior acoustic (*X*_6_), acoustic halo at the peripheral of nodule (*X*_7_), capsule of thyroid (*X*_8_), vascularity (*X*_9_), suspected cervical lymph node metastasis (*X*_10_), elastography score (*X*_11_), hypoenhancement pattern (*X*_12_). The pathological result for the thyroid nodule was the golden standard.

### Statistical analysis

Statistical analyses were performed by R program software package for Windows (R X 64 3.2.3). We used logistic regression to simulate a model to differentiate malignant and benign thyroid nodules. A *p* value <0.05 was considered statistically significant. The statistically significant features were encapsulated in this model. This study got this model’s performance by five folder cross validation, including sensitivity, specificity, accuracy, positive predictive value, and negative predictive value. The receiver operator characteristic curves (ROC) of multivariate observations were drawn, which can assess the logistic regression model’s prediction performance.

## Results

Of the 525 thyroid nodules, 228 (43.4%) were malignant and 297 (56.6%) were benign lesions. All 228 malignant and 27 benign nodules were confirmed by surgery, and the rest of 270 benign were confirmed by cytologic examination. In the group of malignant nodules, the diagnosis included papillary carcinoma (*n* = 220), follicular carcinoma (*n* = 7), and anaplastic carcinoma (*n* = 1). In the group of benign nodules, the diagnosis included nodular goiter (*n* = 208), adenoma (*n* = 70), and Hashimoto’s nodule (*n* = 19). There were 339 persons more than or equal to 40 years old (144 were malignant, and 195 were benign). The remaining 186 persons were less than 40 years old (84 were malignant, and 102 were benign). Clinical data for patients and sonographic characteristics for nodules were presented in Table 1. The gender and age were not found to differ significantly between malignant and benign nodules. Among the sonographic features, calcification, suspected cervical lymph node metastasis, hypoenhancement pattern, margin, shape, vascularity, posterior acoustic, echogenicity, and elastography score had statistic significance between malignant and benign nodules.

**Table 1:** Clinical data for the patients and nodules.

Characteristic	Definition	Total	Benign(*n* = 297)	Malignant(*n* = 228)
Sex				
Women	0	396	228(76.77%)	168(73.68%)
Man	1	129	69(23.23%)	60(26.32%)
Margin				
Irregular margin	1	208	44(14.81%)	164(71.93%)
Regular margin	0	317	253(85.19%)	64(28.07%)
Border				
Ill-defined	1	191	56(18.86%)	135(59.21%)
Well-defined	0	334	241(81.14%)	93(40.79%)
Shape				
Taller-than-wide shape	1	41	3(1.01%)	38(16.67%)
Wider-than-tall shape	0	484	294(98.99%)	190(83.33%)
Echogenicity				
Hypoechogenicity or marked hypoechogenicity	1	69	9(3.03%)	60(26.32%)
No hypoechogenicity or no marked hypoechogenicity	0	456	288(96.97%)	168(73.68%)
Calcification				
Microcalcification	1	214	49(16.50%)	165(72.37%)
No microcalcification	0	311	248(83.50%)	63(27.63%)
Posterior acoustic				
Posterior echo attenuation	1	33	3(1.01%)	30(13.16%)
Posterior echo enhancement or no finding	0	492	294(98.99%)	198(86.84%)
Peripheral acoustic halo				
Present	1	70	55(18.52%)	15(6.58%)
Absent	0	455	242(81.48%)	213(93.42%)
Capsule of thyroid				
Interruption	1	29	4(1.35%)	25(10.96%)
Complete	0	496	293(98.65%)	203(89.04%)
Vascularity				
Central vascularity	1	267	103(34.68%)	164(71.93%)
Without central vascularity	0	258	194(65.32%)	64(28.07%)
Suspected cervical lymph node metastasis				
Present	1	92	2(0.67%)	90(39.47%)
Absent	0	433	295(99.32%)	138(60.53%)
Elastography score				
≥ 4	1	81	12(4.04%)	69(30.26%)
<4	0	444	285(95.96%)	159(69.74%)
Hypoenhancement pattern				
Heterogeneous hypo-enhancement pattern	1	285	185(62.29%)	100(43.86%)
No heterogeneous hypo-enhancement pattern	0	240	112(37.71%)	128(56.14%)

The summary after logistic regression analysis about every sonographic feature was detailed in the Table 2. The fitted model was established:

$$\begin{array}{ccc} Z & = & sigmoid(\chi )\\ & = & 1/(1+e^{-(-1.84+1.21X_{1}+2.55X_{3}+1.23X_{4}+1.73X_{5}+1.91X_{6}+0.92X_{9}+3.91X_{10}+0.98X_{11}-1.16X_{12})}) \end{array}$$

where *Z* was the probability of suffering from maligant thyroid nodule, *sigmoid*(⋅) was the activation function of logistic regression,

$$\begin{array}{c} sigmoid\left(\chi \right)=1/\left(1+e^{-\chi }\right) \end{array}$$

**Fig 1:**
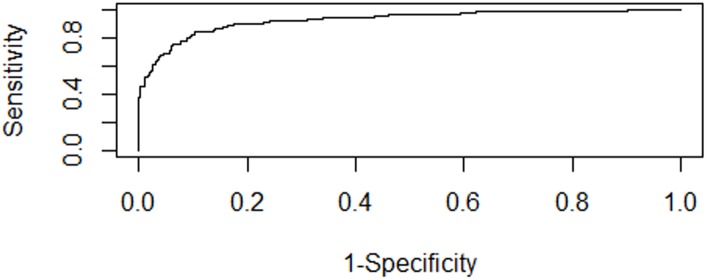
The ROC curve of logistic regression.

*χ* denoted the all features. The thyroid nodules were regarded as malignant if *Z* was equal to or greater than 0.5. In contrast, the thyroid nodules were regarded as benign if *Z* was less than 0.5. After five folder cross validation, we also got the receiver operator characteristic curve (Fig 1) which is close to top left corner of the coordinate system. The area under the ROC curve (AUC) for using this formula to differentiate malignant and benign thyroid nodules was 0.930. The sensitivity, specificity, accuracy, positive predictive value, and negative predictive value were 83.77%, 89.56%, 87.05%, 86.04%, and 87.79% respectively.

**Table 2:** The result of logistic regression analysis.

Characteristic	*Label*	*Estimatestd*	*Error*	*z* *value*	*Pr*(> |*z*|)
Intercept		-1.837	0.649	-2.832	0.0046**
Age		-0.012	0.013	-0.918	0.3584
Sex		0.128	0.350	0.364	0.7157
Margin	*X*_1_	1.214	0.349	3.477	0.0005***
Border	*X*_2_	-0.067	0.368	-0.183	0.8548
Shape	*X*_3_	2.551	0.746	3.422	0.0006***
Echogenicity	*X*_4_	1.233	0.524	2.354	0.0186*
Calcification	*X*_5_	1.728	0.317	5.453	4.95e-08***
Posterior acoustic	*X*_6_	1.914	0.727	2.632	0.0085**
Peripheral acoustic halo	*X*_7_	-0.325	0.453	-0.718	0.4729
Capsule of thyroid	*X*_8_	0.925	0.772	1.198	0.2309
Vascularity	*X*_9_	0.916	0.311	2.945	0.0032**
Suspected cervical lymph node metastasis	*X*_10_	3.909	0.784	4.989	6.07e-07***
Elastography score	*X*_11_	0.978	0.455	2.147	0.0318*
Hypoenhancement pattern	*X*_12_	-1.162	0.305	-3.812	0.0001***

The asterisk designates the statistically significant features if *p* value is less than 0.05.

0 ‘***’ 0.001 ‘**’ 0.01 ‘*’

## Discussion

Nowadays thyroid nodules have a high incidence because of the widespread use of high-resolution ultrasound in clinical practice. Most of thyroid nodules are benign, whereas it is uneasy to find the thyroid carcinomas because of the hidden early clinical symptoms of them. Pathological findings are the gold standards in differentiating benign and malignant thyroid nodules. High-frequency ultrasound plays an important role in the diagnosis of differentiating malignant from benign nodules because of its economy, noninvasive, and convenient features. Known suspicious US features of malignant thyroid nodules are irregular margins, ill-defined border, hypoechogenicity or marked hypoechogenicity, microcalcification, taller-than-wide shape and so on. Based on the fact that malignant are harder than benign nodules, ultrasound elastography is a reliable technique in the differentiation of malignant and benign thyroid nodules [11, 12]. On the basis of previous study, thyroid nodules with the ARFI-induced SE score ≥ 4 would be regarded as malignancy. CEUS is a useful screening tool to differentiate thyroid nodules. If the thyroid nodules are malignant, lesions could be heterogeneous hypoenhanced after the infusion of contrast agent. In clinical practice, no single feature can authentically predict the risk of malignant. However, a combination of suspicious ultrasound features is known to provide better diagnostic accuracy.

Suspicious features reported in different studies vary slightly. Chng et al. [9] reported that nodules with irregular margin, hypoechogenicity, and taller-than-wide morphology significantly had higher percentages of malignancy comparing to benign. Kim et al. [13] reported that hypoechogenicity, microcalcifications, infiltrative margin, solid composition and taller-than-wide shape were significant features associated with malignancy. Tay et al. [14] investigated that there was not significantly different in size of nodules, patients’ gender and age. In the present study, we got the consistent result with them. Sex (*p* = 0.698) and age (*p* = 0.213) did not have significant difference between benign and malignant groups. They also found that benign nodules have all four US characteristics: well-defined margin, no calcification, no increase in vascularity, and no suspected cervical lymph node metastasis. Shweel et al. [15] found that elastography alone, as well as the combination of elastography and conventional US, showed superior diagnostic performance when compared with conventional US examination. Zhao et al. [16] reported that CEUS in combination with conventional US could improve the diagnostic accuracy of sub-centimeter nodules and reduced unnecessary surgery.

In daily practical work, it is common that not all malignant have suspicious ultrasound features of malignancy. In other words, it is hard for clinicians to determine whether the nodule is malignant or not when the lesion don’t have all suspicious features. In this study, we used the above mentioned 12 features in Table 1 to select the significant features and simulate a model to diagnose thyroid nodules. In the logistic regression model, the significant features which *p* values less than 0.05 were showed in Table 2 (including margin, shape, echogenicity, calcification, posterior acoustic, vascularity, suspected cervical lymph node metastasis, elastography score, and hypoenhancement). Malignant nodules may show irregular margin because of irregular growth of the fibrous stroma surrounding the carcinoma or carcinoma invasion into surrounding thyroid parenchyma. The taller-than-wide shape was first proposed by Kim et al [17], which was based on the fact that growth of most benign nodules has been found to remain within normal tissue planes, whereas malignant nodules grow across normal tissue planes. Because of the uneven intranodular composition, malignant nodules may display hypoechogenicity or marked hypoechogenicity, and posterior echo attenuation. Microcalcification was thought to be the representation of calcified psammoma bodies of carcinoma. The rapid growth and hyperplasia reaction of thyroid carcinoma cells may result in microcalcification. The second explanation for microcalcification is the secretion of glycoproteins and mucopolysaccharides by carcinoma cells. It is reported that most thyroid cancers showed increased vascularity. Thyroid carcinoma cells could produce angiogenic factors, thereby promoting the formation of neovascularization. In the study of Papini [18], suspected cervical lymph node metastasis was the most frequent finding in thyroid carcinoma. A published study reported that ARFI elastography seemed to be a valuable technique to differentiate thyroid carcinomas from benign lesions. Regarding CEUS, a small number of papers have documented that the diagnostic accuracy of CEUS following intravenous microbubble contrast. The tortuosity and irregularity of newly formed vessels in malignant lesions may lead to heterogeneous hypo-enhancement pattern. Although the presence of ill-defined border, acoustic halo and interrupted capsule of thyroid were found to be predictive of malignancy in some studies [19, 20]. However, these three characteristics were also not significant in this study. It is probably because some benign thyroid nodules have ill-defined border, such as subacute thyroiditis and Hashimoto’s thyroiditis. Acoustic halo may be due to the presence of edema which may appear in benign nodules. The presence of interrupted capsule of thyroid was not significantly associated with malignancy. It was probably related to that some nodules are just protrusions but without breakthrough.

**Fig 2:**
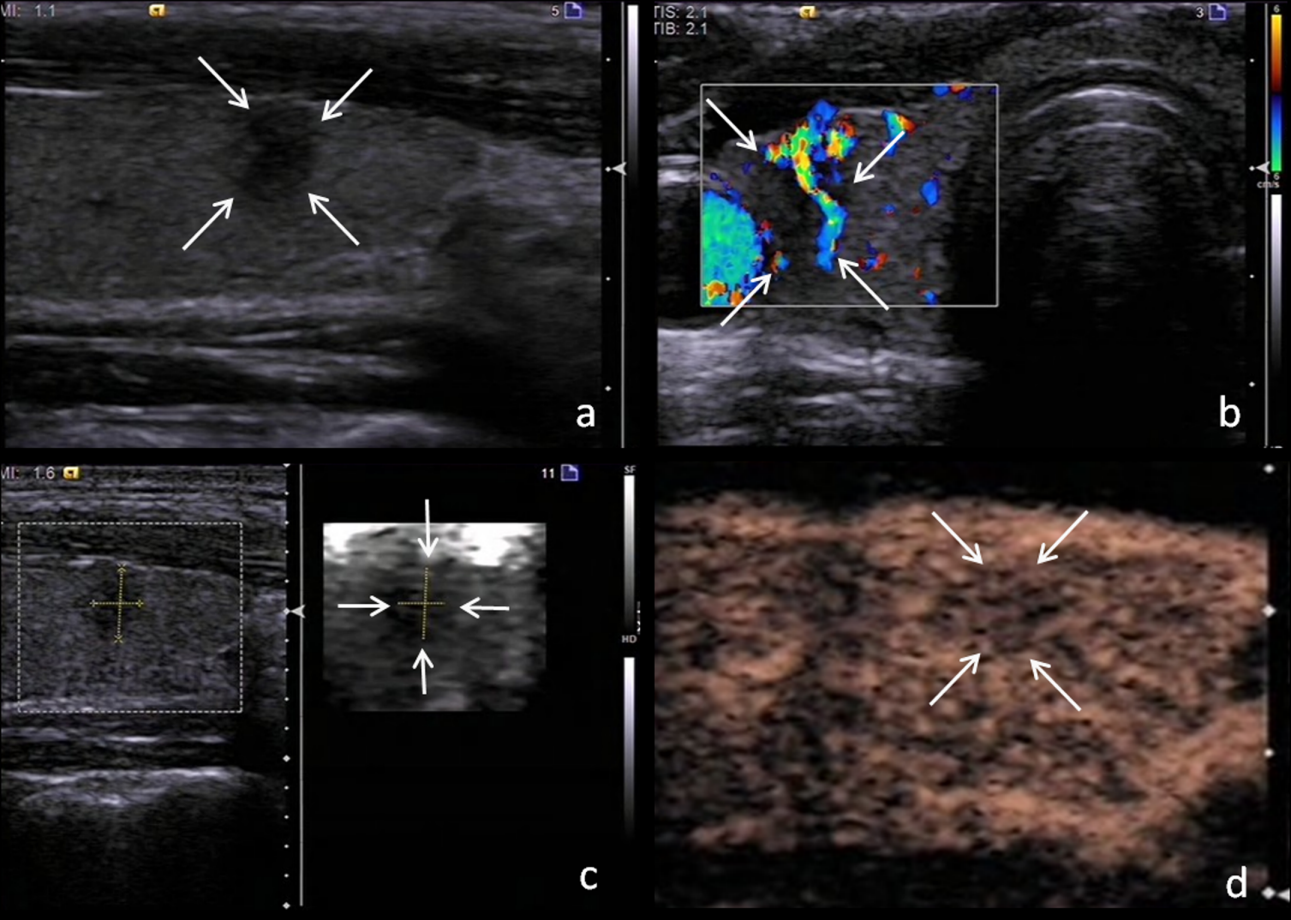
Ultrasonography images of a malignant thyroid nodule in the right lobe of a 44-year-old woman. The calculated predictive value with the logistic regression formula was 0.980. Surgical pathology proved a papillary thyroid carcinoma. (a) Conventional US image showing a thyroid nodule with irregular margin, ill-defined border, taller-than-wide shape, hypoechogenicity in the longitudinal section. (b) Color Doppler US image showing central vascularity in the transversal section. (c) SE image showing elastography score of 4 in the longitudinal section. (d) CEUS image showing a heterogeneous mass with hypo-enhancement pattern in the longitudinal section.

**Fig 3:**
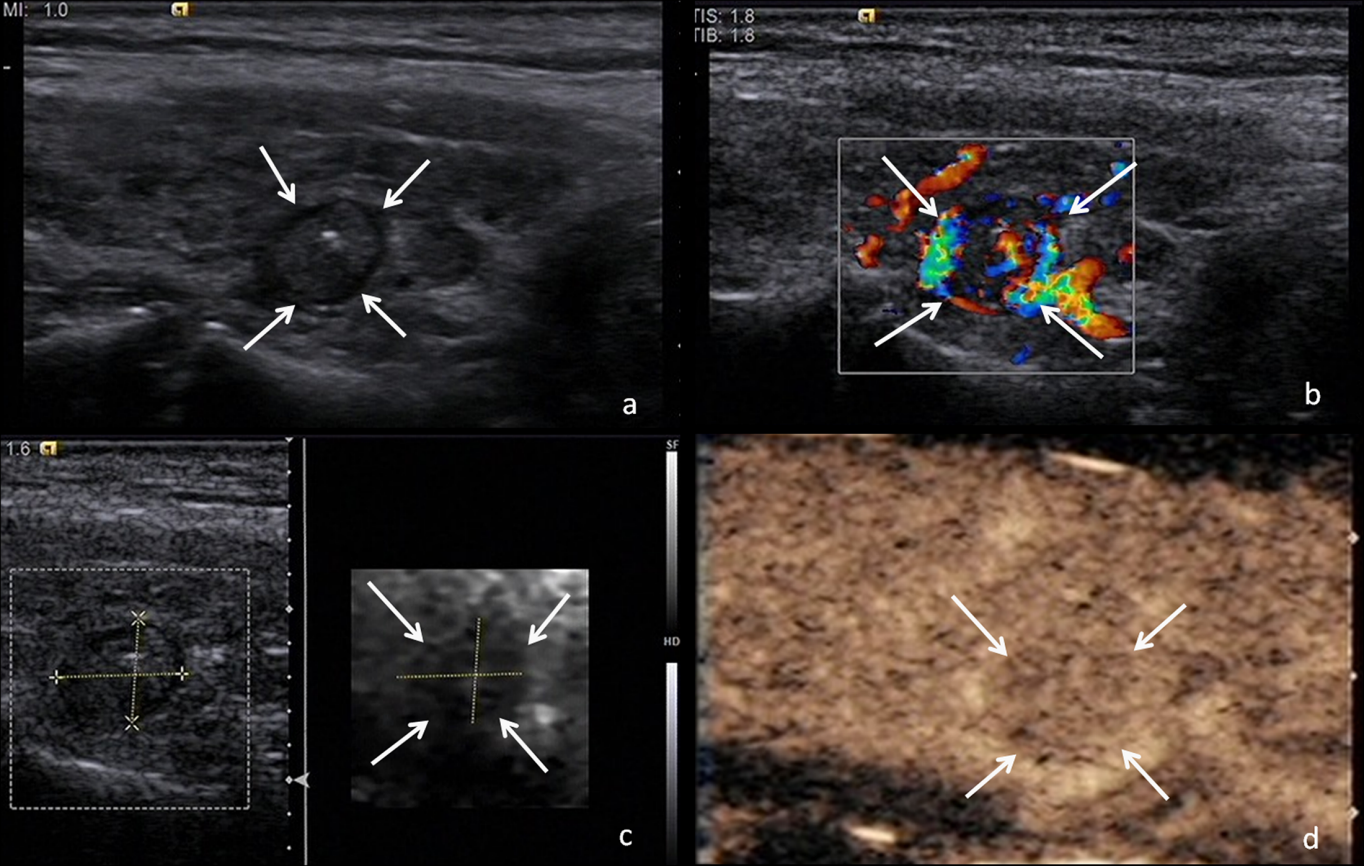
Ultrasonography images of a benign thyroid nodule in the right lobe of a 50-year-old woman. The calculated predictive value with the logistic regression formula was 0.412. Surgical pathology proved a nodular goiter. (a) Conventional US image showing a thyroid nodule with microcalcification, and peripheral acoustic halo in the longitudinal section. (b) Color Doppler US image showing presence of central vascularity in the longitudinal section. (c) SE image showing elastography score of 3 in the longitudinal section. (d) CEUS image showing a heterogeneous mass with hypo-enhancement pattern in the longitudinal section.

In this study, one nodule was found to have irregular margin, ill-defined border, taller-than-wide shape, hypoechogenicity, central vascularity, elastography score of 4 and heterogeneous hypo-enhancement. The predictive value calculated with the logistic regression formula, 0.974, supported the presence of a malignant nodule. Pathologic analysis after surgery revealed a papillary thyroid carcinoma (Fig 2). The other nodule was found to have microcalcification, and peripheral acoustic halo, central vascularity, elastography score of 3 and heterogeneous hypo-enhancement. The predictive value calculated with the logistic regression formula, 0.350, supported the presence of a benign nodule. Pathologic analysis after surgery revealed a nodular goiter (Fig 3).

The strength of this study is that we used 12 features regarding SE, CEUS in combination with conventional US to screen the significant features in the diagnosis of malignancy and benign. In addition, the established prediction model in this study is simple and convenient, allowing a widespread use among clinicians. There are several limitations in this study. First, we have not considered the size of the nodules and the number of nodules. Second, the sample size was limited in this study. And this was a retrospective study. The established model needs more researches to validate and support. We are looking forward to perform large-sample study. Third, we did not analyze the variation of interobserver and intraobserver. We are looking forward to performing the analysis of interobserver and intraobserver variation in future.

## Conclusion

The logistic regression model for significant sonographic features of conventional US, SE, and CEUS is an effective and accurate diagnostic tool for differentiating malignant and benign thyroid nodules. Independent risk features for determination are calcification, suspected cervical lymph node metastasis, hypoenhancement pattern, margin, shape, vascularity, posterior acoustic, echogenicity, and elastography score. Elastography and CEUS show a highly valuable performance in the diagnostic approach of thyroid nodules.

## Supporting information

S1 Data Data of thyroid nodules. (XLSX)
